# Construction of 3D bioprinting of HAP/collagen scaffold in gelation bath for bone tissue engineering

**DOI:** 10.1093/rb/rbad067

**Published:** 2023-08-11

**Authors:** Chuang Guo, Jiacheng Wu, Yiming Zeng, Hong Li

**Affiliations:** Department of Materials Science and Engneering, College of Chemistry and Materials Science, Jinan University, Guangzhou, Guangdong 511436, China; Ministry of Education, Engineering Centre of Artificial Organs and Materials, Guangzhou, Guangdong 510632, China; Department of Materials Science and Engneering, College of Chemistry and Materials Science, Jinan University, Guangzhou, Guangdong 511436, China; Ministry of Education, Engineering Centre of Artificial Organs and Materials, Guangzhou, Guangdong 510632, China; Department of Materials Science and Engneering, College of Chemistry and Materials Science, Jinan University, Guangzhou, Guangdong 511436, China; Ministry of Education, Engineering Centre of Artificial Organs and Materials, Guangzhou, Guangdong 510632, China; Department of Materials Science and Engneering, College of Chemistry and Materials Science, Jinan University, Guangzhou, Guangdong 511436, China; Ministry of Education, Engineering Centre of Artificial Organs and Materials, Guangzhou, Guangdong 510632, China

**Keywords:** 3D printing, collagen, hydroxyapatite, scaffold, gelation bath

## Abstract

Reconstruction of bone defects remains a clinical challenge, and 3D bioprinting is a fabrication technology to treat it via tissue engineering. Collagen is currently the most popular cell scaffold for tissue engineering; however, a shortage of printability and low mechanical strength limited its application via 3D bioprinting. In the study, aiding with a gelatin support bath, a collagen-based scaffold was fabricated via 3D printing, where hydroxyapatite (HAP) and bone marrow mesenchymal stem cells (BMSCs) were added to mimic the composition of bone. The results showed that the blend of HAP and collagen showed suitable rheological performance for 3D extrusion printing and enhanced the composite scaffold’s strength. The gelatin support bath could effectively support the HAP/collagen scaffold’s dimension with designed patterns at room temperature. BMSCs in/on the scaffold kept living and proliferating, and there was a high alkaline phosphate expression. The printed collagen-based scaffold with biocompatibility, mechanical properties and bioactivity provides a new way for bone tissue engineering via 3D bioprinting.

## Graphical abstract

**Figure:**
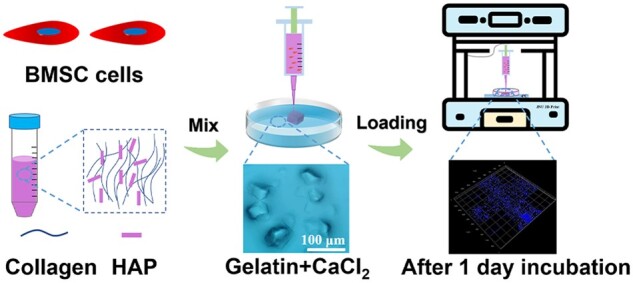

## Introduction

Bone defects are common and frequently occurring diseases. Although bone has a certain capability for regeneration and self-repair, sizeable segmental bone defects caused by severe trauma, tumor resection, cancer or congenital diseases cannot be self-repaired effectively [1]. Every year, more than 2.2 million people worldwide require bone-graft procedures to repair bone defects. With the advent of an aging society, a massive market of bone grafts has been breeding.

Previous therapeutic approaches such as autografts, allografts and xenografts have been restricted due to associated drawbacks such as limited donor supply and donor sites, the potential risk of disease transmission and immune response after implantation [2]. Due to these limitations associated with natural bone grafts, bone tissue engineering (BTE) has drawn significant attention to creating novel constructs to restore, maintain and improve bone function. BTE generally requires the use of a porous scaffold [3], which serves as a 3D (three-dimensional) structure providing a temporary environment for mechanical support to cell proliferation, extracellular matrix formation, cellular activity, oxygen diffusion, nutrient delivery and waste removal [4]. Therefore, a porous matrix that mimics the tissue’s living milieu characteristics should be used to create the scaffold to increase the procedure’s success.

Conventional scaffold fabrication methods, such as solvent-casting [5] and freeze-drying [6], have had limited capacity to control pore size, pore geometry, pore interconnectivity and the spatial distribution of pores in scaffolds [7, 8]. Compared to previous methods of scaffold manufacturing, 3D printing is considered the most promising technique for fabricating biomedical scaffolds [9], artificial tissues [10] and organs [11] due to its enhanced ability in controlling scaffold structure [12]. Moreover, recent developments in 3D printing technology have also enabled the incorporation of living cells and growth factors into scaffolds during fabrication [13, 14], and a denoted bioprinting approach has been used to reconstruct tissue. Anyway, a scaffold material that can support cell loading with good printability and biocompatibility is still in shortage, which limits the application of bioprinting for tissue repair.

As the main components of bone tissue, collagen and hydroxyapatite (HAP) are often used as the ideal materials to construct bone tissue repair scaffolds [15–17]. However, whether or not the mixture of collagen and HAP is challenging to fabricate via 3D printing [18]. In previous studies, the way by the low-temperature freezing scheme of 3D printing was applied to print bone repair scaffolds, using HAP and collagen as extrusion materials [19]. When the materials arrived at low temperatures, the print platform momentarily froze, keeping the shape of the scaffold, solving the problem that was not easy to process. The scaffold materials are close to the bone’s composition and show potential for bone tissue repair [20–22]. However, the low-temperature freezing printing process requires the addition of lubricant and expensive equipment, as well as a demanding processing environment and complicated procedures [23]. Some studies have also used glutaraldehyde as a crosslinking agent to synchronously generate HAP deposition and collagen crosslinking to avoid a low-temperature environment [24, 25]. However, the introduction of the crosslinking agents might bring specific cytotoxicity to the materials [23], which must be removed later [26–28]. Also, these two methods cannot achieve printing with living cells. Further, the timing of cell proliferation is crucial for tissue repair. Therefore, the scaffold should replicate the physiological state of native cells and maintain their *in vivo* functionality. Moreover, a way to realize the printability and cell viability of the BTE scaffold still is a challenge now [29, 30].

Near recently, with its temperature-sensitive properties, a gelatin support bath has been proven to assist in the printing molding of collagen materials at room temperature [11, 31, 32], which may bring a possibility to bioprinting collagen hydrogel with cell-laden at room temperature. Similarly, nanoparticle-enhanced hydrogel bio-ink can improve the materials’ printing performance, rheological properties and mechanical properties [33]. However, there is a lack of a high-fidelity method for constructing macroscopic ordered 3D bone tissue scaffolds in a cell-friendly environment. In this study, we attempted to explore 3D bioprinting processing technology at a suitable temperature with the help of a gelatin support bath. The designed 3D printed scaffold has a similar composition to the main components in bone tissue, which also realized the cell-laden due to the mild processing environment. The study explores one of the effective ways to use the bio-manufacturing process for bone repair.

## Materials and methods

### Preparation and characterization of composite

To simulate the composition of natural bone. A certain amount of collagen (extraction from rat tail tendon by enzymatic acid method [34]) was dissolved in 0.5 M of acetic acid and placed in a 4°C refrigerator for 12 h until the collagen sponge was completely dissolved. At the same time, a certain amount of HAP (synthesized by hydrothermal method [35]) was dispersed in a potassium hydroxide solution of 1 M, followed by ultrasonic treatment for 20 min and then stirred for 12 h at room temperature to obtain HAP suspension. The collagen solution was combined with the HAP suspension to achieve a final mass ratio of 35 and 65 wt% for collagen and HAP, respectively [36]. Additionally, the final concentration of collagen reached 60 mg/ml. (To prepare 3 ml of printing materials, firstly, weigh 180 mg of collagen sponge and dissolve it in 2 ml of 0.5 M acetic acid solution. Refrigerate the collagen solution at 4°C until it is completely dissolved. Next, weigh out 334.3 mg of HAP and mix it with 1 ml of a potassium oxide solution (1 M) to create a suspension. Finally, thoroughly stir the collagen and HAP suspension at 4°C until achieving homogenous mixing while continuously monitoring and adjusting the pH value.) This process was carried out in a 4°C aseptic environment to avoid collagen denaturation. The prepared sterile print composite was placed in a 4°C refrigerator and mixed with 2 × 10^6^ bone marrow mesenchymal stem cells (BMSCs) suspension per milliliter in Dulbecco’s modified eagle medium (DMEM) for cell-laden printing. The collagen concentration in the printed materials containing the cells was 50 mg/ml. Typically, the bio-ink was centrifuged to remove bubbles.

A Kinexus Pro rheometer (Malvern, UK) was used to measure the rheological behavior of the composite. The steady-state shearing of the pastes was evaluated by scanning within the 0.01–1000/s shear rate range at 4°C. Moreover, the viscosity recovery was measured by a three-step shear scanning experiment, with a duration of 60 s for each stage and shear stress varying between 10^−4^ and 10^−3^ MPa [37].

To test that collagen and HAP were not denatured or destroyed during the preparation. Moreover, the composite was tested by an X-ray diffractometer (XRD) (Miniflex 600, Japan) and Fourier transform infrared spectrometer (FTIR) (PerkinElmer, USA). The particle size and surface potential of HAP were observed using Nanometrics (Zetasizer Nano ZSE, Malvern), while the morphology of HAP before and after mixing was observed by transmission electron microscope (TEM) (JEOL/JEM-1400 Flash, Japan).

### Extrusion 3D printing

The 3D models (Supplementary Fig. S1) were designed and created by Creo (Parametric Technology Corporation Creo Parametric 5.01.0) software, saved as StereoLithography (STL) files and then sliced in the Cura (Ultimaker, V15.06) software to generate a printing scheme. The extruded 3D printer was modified from a melt printer (ZD-2000A, China). The nozzle of the fusion printer was replaced with a syringe, which was controlled by a conveyor belt.

### Three-dimensional bioprinting

#### Printing process in gelatin support bath

The gelatin support bath was prepared according to the support information. The 3D printer is equipped with a semiconductor refrigeration sheet, and the insulation temperature of the semiconductor refrigeration sheet is set to 4°C. The gelatin support bath is removed from the 4°C refrigerator and placed above the semiconductor cooling sheet when printing the scaffold. The semiconductor cooling sheet provides continuous cooling to the gelatin support bath, maintaining its temperature at 4°C. Meanwhile, the composite materials with or without cell-laden were added to the syringe and then fixed on the injector on the specified frame. The 3D printer was started according to the designed program. After printing, the printed scaffold was placed in an ammonia atmosphere to regulate pH and gelatinize the collagen for 5 min. Then, it was placed in a 37°C thermostatic water bath to incubate for at least 1 h to melt the gelatin and further gelatinize the collagen. The scaffolds were taken out and washed in sterile water, repeated two to three times, and then the scaffolds were soaked in phosphate buffer saline (PBS) or DMEM and stored in a 4°C refrigerator.

#### Characterization

The scaffolds were observed under the scanning electron microscope (SEM) (FlexSEM1000 II, Hitachi High-Tech Corporation, Japan). The energy dispersive spectrometer was used to analyze the elements in the scaffolds and observe the distribution of elements in the scaffolds.

The dry and wet scaffolds were mechanically tested using a universal testing machine (Shimjin universal testing machine AGS-X10KN, Japan) with a 0.5 mm/min compression speed and a strain of 80% for each sample. Set up five parallel samples.

### Cell culture

BMSCs originated from rats, and BMSCs were cultured in DMEM with 10% fetal bovine serum and 1% penicillin/streptomycin in a humidified atmosphere of 37°C with 5% CO_2_. The culture media were changed every 2 days, and the cells were passaged by trypsin after reaching 80% confluence.

The cell-laden scaffolds were printed in a sterile environment suitable for cell survival. After removing the gelatin support bath, the printed scaffold was placed into a 12-well plate. DMEM medium was added to the well plate and cultured in an incubator at 37°C and replace the DMEM medium once a day.

#### Cytotoxicity

The scaffolds were soaked in PBS and sterilized by UV irradiation for 2 h. Then, the BMSCs suspension was seeded on the scaffolds at 4 × 10^4^ cells per well, and 1 ml medium was added. After 1, 4 and 7 days of culture, cell counting kit-8 (CCK-8) assay and AM (acetyl methoxymethyl)/PI (propidium iodide) live/dead assay was used to evaluate the cytotoxicity of scaffolds. When measuring the cell viability by CCK-8 assay, the scaffolds were transferred into a new well plate. All studies were carried out in triplicate.

The cytoskeletal actin and nucleus of the BMSCs after 24 and 48 h of culturing on the scaffolds were stained with 4′,6-diamidino-2-phenylindole (DAPI), and rhodamine-phalloidin following the manual and photographed under a laser scanning confocal microscope (LSCM) (LSM 880 with AiryScan, Carl Zeiss).

#### Osteogenic ability evaluation

Alkaline phosphatase (ALP) and bicinchoninic acid protein kits were used to test the osteogenic ability of the scaffolds. After BMSCs were cultured on scaffolds for 7 and 14 days, the scaffolds were processed according to the instructions, and the optical density (OD) values of each well were determined using an Automatic Elisa analyzer (Cytation 3, USA). At last, the content of ALP was calculated according to the formula in the manual. The experiment was carried out in triplicates.

### Cells in 3D printed cell-laden scaffold

To observe the distribution of cells in the scaffolds. The cell-laden composite scaffolds were taken out after incubation for 1 day. And then, stained with DAPI according to the instructions, the scaffolds were placed in a laser confocal dish and observed and photographed under an LSCM (LSM 880 with AiryScan, Carl Zeiss).

### Statistical analysis

In this study, each experiment was repeated three times, and the data of each study were expressed as the mean ± standard deviation (SD). All results were statistically analyzed by one-way analysis of variance (ANOVA). The difference was considered to be statistically significant when *P* < 0.05.

## Results and discussion

### Composition and structural analysis

**Figure 1.**
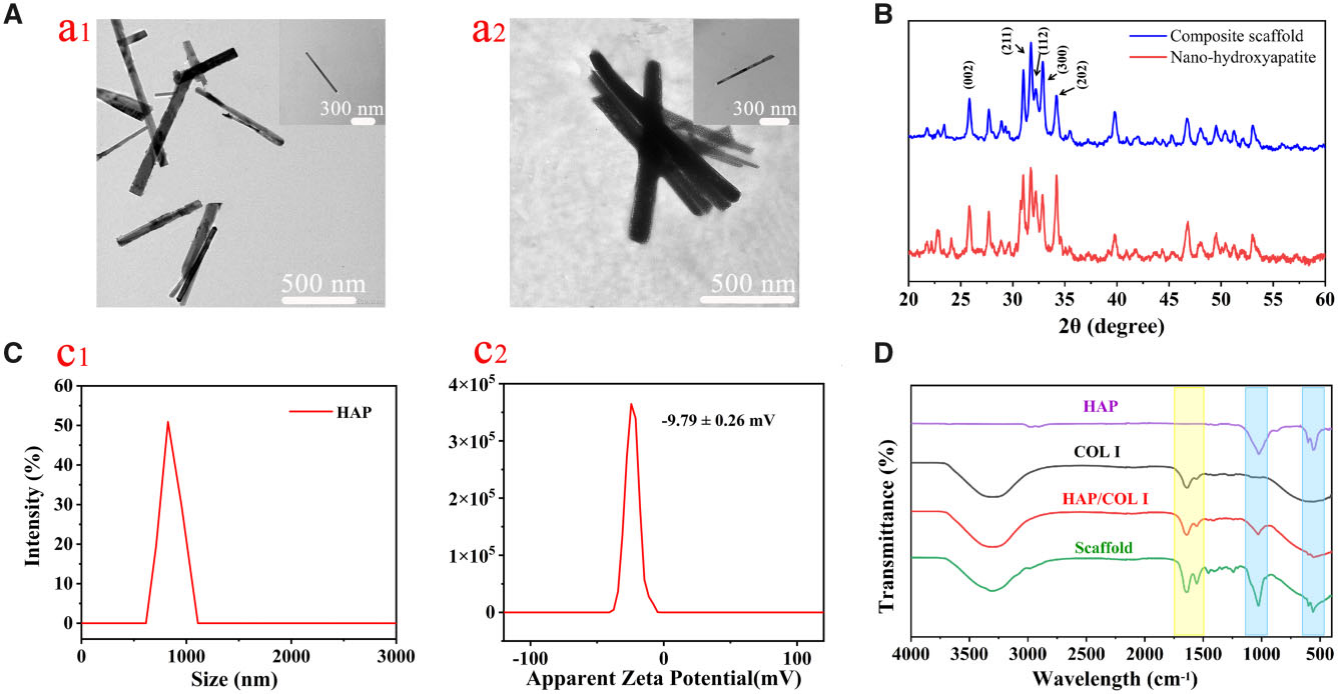
Characterization of collagen and HAP: (**A**) TEM images of HAP powder and composite materials; (**B**) XRD patterns of HAP and composite materials; (**C**) particle size and zeta potential of HAP powder; (**D**) FTIR spectra.

Figure 1a_1_ is the TEM of HAP crystals, which shows that HAP is a nano rod-like structure with a length of about 800 nm. The rod-like HAP crystals in the composite are shown in Figure 1a_2_. It can be seen that the morphology of HAP did not change during the preparation process. Figure 1B is the XRD analysis pattern of the samples. The characteristic peaks of the composite and HAP powder are consistent, corresponding to the (002), (211), (112), (300) and (202) crystal planes matching with JCPDS09-0432 of standard HAP, respectively, which further illustrates that the HAP maintains its original phase during the preparation of the composite. Figure 1c_1_ is the particle size distribution of self-made HAP powder. The HAP particle size of the rod-like structure is concentrated between 800 and 1000 nm, indicating that the particle size of the self-made HAP is uniform. Figure 1c_2_ shows the Zeta potential measurement of HAP. It is shown that the surface of HAP is negatively charged, and the measured display value is −9.79 ± 0.26 mV. The intact three-stranded helical structure of collagen is indicated by the peak at 1454–1240/cm, corresponding to the proline–glycine–hydroxyproline (PRO–GLY–HYP) signature sequence in Figure 1D. Additionally, characteristic peaks of HAP’s ${\text{PO}}_{4}^{3-}$, observed at 1023 and 556/cm, were identified [38].

**Figure 2.**
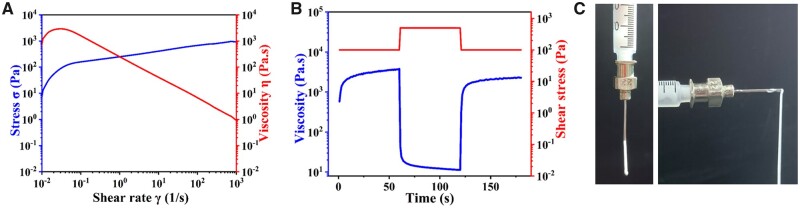
Rheological analyses of the composite materials: (**A**) shear thinning at 4°C; (**B**) viscosity recovery; (**C**) extrusion state of composite materials at 4°C.

The obtained composite structure agrees with the composite of HAP/collagen. In the composite system, the crucial interaction between the two materials is the electrostatic interaction and hydrogen bond between the components. Studies have shown that hydrogen bonds exist between collagen and HAP [39, 40]. Meanwhile, collagen has a positive potential when the pH is between 6 and 8 [41]. The HAP used in the study had a negative potential, which can achieve electrostatic interaction with the collagen with a positive potential. The interaction may bring a stable composite, which may benefit further printing.

### Rheological properties

The shear thinning behavior of the composite was investigated at 4°C, as shown in Figure 2A. With the increase in both shear stress and rate, the viscosity of the materials initially exhibited an upward trend. The viscosity immediately decreased as the shear rate increased, eventually reaching a maximum value of 0.84 ± 0.05 Pa·s. Figure 2B shows the recovery behavior of sample viscosity at 4°C. The initial viscosity was measured at 3017 ± 136.1 Pa·s for the first 60 s, followed by a sudden decrease to 11.6 ± 0.88 Pa·s with an increase in shear stress during the subsequent 60 s. After the third 60-s interval, the viscosity of the composites recovered to 2441.6 ± 186.1 Pa·s. The studies indicate that the rapid recovery of viscosity in the material post-extrusion contributes to enhancing the fidelity of printed scaffolds [42, 43]. Figure 2C shows the composite extruded from a syringe at 4°C. The materials can be extruded in continuous filaments, indicating that the composites have good fluidity.

The previous study has demonstrated that collagen undergoes stretching and elongation under the influence of molecular chain force after being subjected to shearing stress [37], which reduces intermolecular entanglement, promotes sliding and decreases viscosity. The phenomenon corresponds to the viscosity during 60–120 s. In a stage of viscosity reduction, the molecular chains are re-entangled after the shear force is reduced, hindering the sliding and causing the viscosity to rise again at the following time. In this process, a loss of 17% was irreversible [37], which also keeps in accord with our result.

### Printability in a support bath

**Figure 3.**
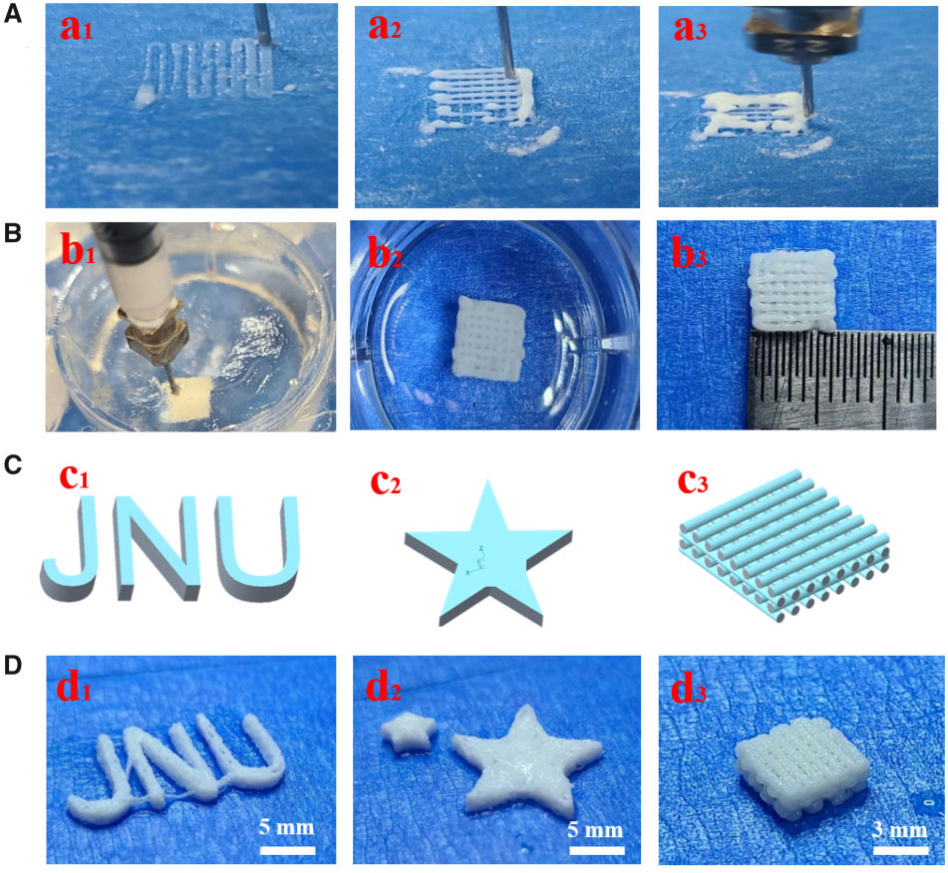
The 3D printing process includes: (**A**) printing in mid-air; (**B**) printing with support from a gelatin bath; (**C**) designing 3D models and (**D**) producing printed 3D models.

In Figure 3A, printing was carried out without using a gelatin support bath. As depicted in Figure 3a_1_, the initial layer was printed with precision, accurately reproducing the topography of the scaffolds. During the printing of the second and third layers (a_2_, a_3_), the printed patterns failed to align with their intended topographies and instead merged with the first layer, resulting in an inability to realize the designed 3D model and ultimately causing the structural collapse of the scaffolds. Figure 3B shows that the composite was printed in a gelatin support bath. The scaffolds were constructed according to the design of the 3D model. As shown in Figure 3C and D, with the aid of the gelatin support bath, the designed patterns of ‘JNU’, ‘Pentagram’ and ‘wood-piled scaffolds’ were printed, respectively. The results indicate that the gelatin support bath effectively sustains the composite gel, preventing structural collapse during printing and facilitating the creation of 3D structures. According to the previous report [7], the gelatin support bath has the characteristics of Bingham plastic fluid, which behaves as a rigid body under low shear force. Under higher shear force, it exhibits the properties of a viscous fluid, allowing for seamless extrusion from printer nozzles and maintenance of pattern integrity during printing. From the aforementioned results, it can be inferred that the gelatin support bath serves as an optimal foundation for constructing desired geometries with printed gel materials. Additionally, the composite gel exhibits exceptional printability at ambient temperatures.

### Morphology of scaffold

**Figure 4.**
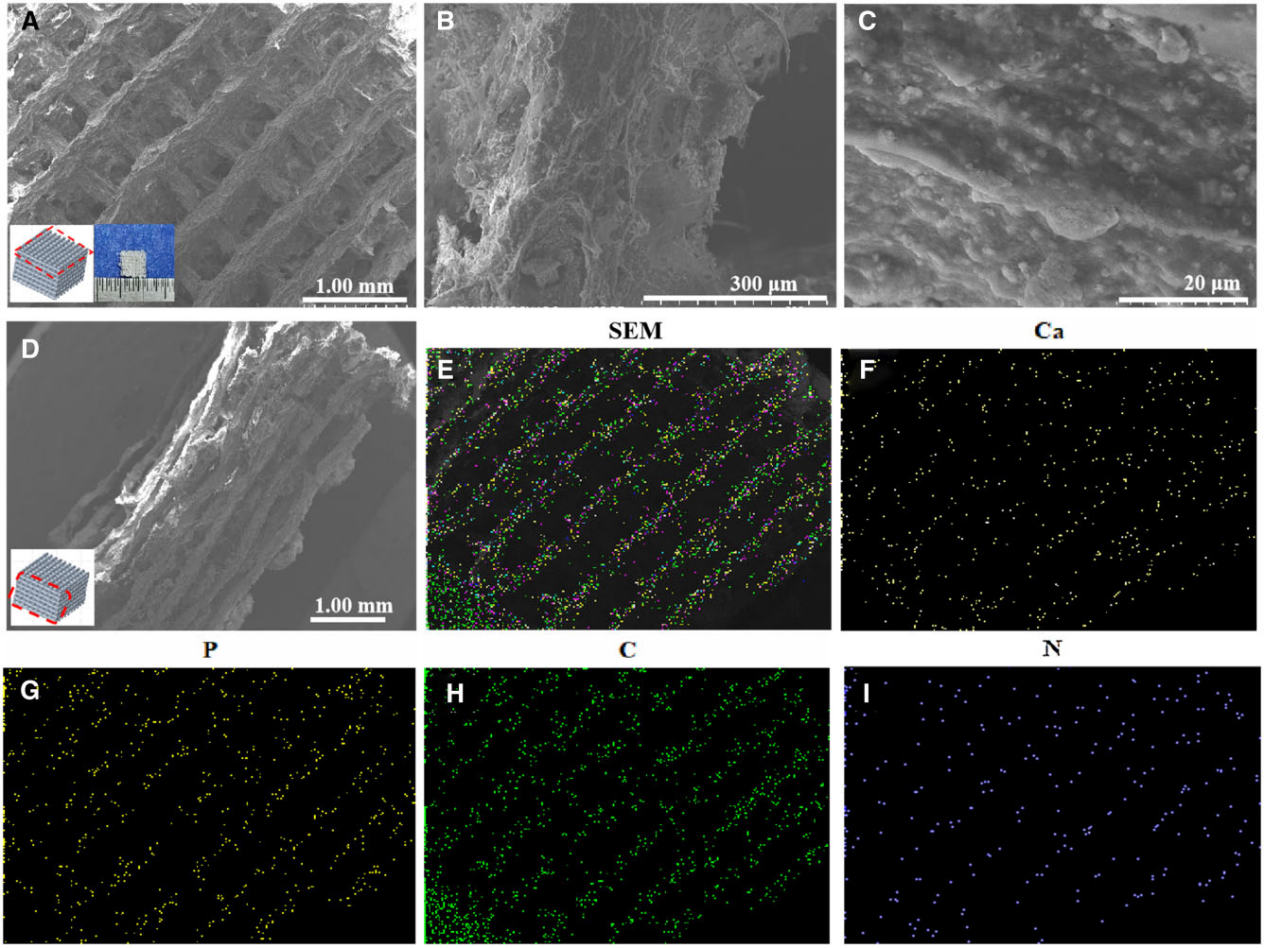
SEM Images and element distribution of lyophilized scaffolds: (**A**–**C**) top-view SEM images of the scaffold in different magnifications; (**D**) side-view SEM image of the scaffold; (**E**) energy spectrum of the scaffold, corresponding to image (A); (**F**–**I**) the distribution of Ca, P, C and N elements in the scaffold, respectively.

Figure 4A–D shows the morphology of the printed scaffold after freeze-drying, as observed by SEM. The freeze-dried scaffold maintained a porous interconnectivity structure with an overall orderly arrangement in 3D, as shown in Figure 4A and D, which should be conducive to transporting and exchanging nutrients. The pore with the size of about 500 × 800 μm, as we know, it benefits the proliferation of cells [44]. At the same time, it can be seen that the pore surface of the scaffolds was rough and uneven (Figure 4B), which would be beneficial to the adhesion and migration of cells [37]. High magnification imaging revealed uniform dispersion of HAP particles within the collagen matrix, as depicted in Figure 4C. The scaffolds’ outline was discernible from energy spectrum analysis, as shown in Figure 4E. Moreover, the homogenous distribution of Ca, P, C and N elements confirms HAP crystals in the collagen matrix in Figure 4F–I.

### Mechanical properties

**Figure 5.**
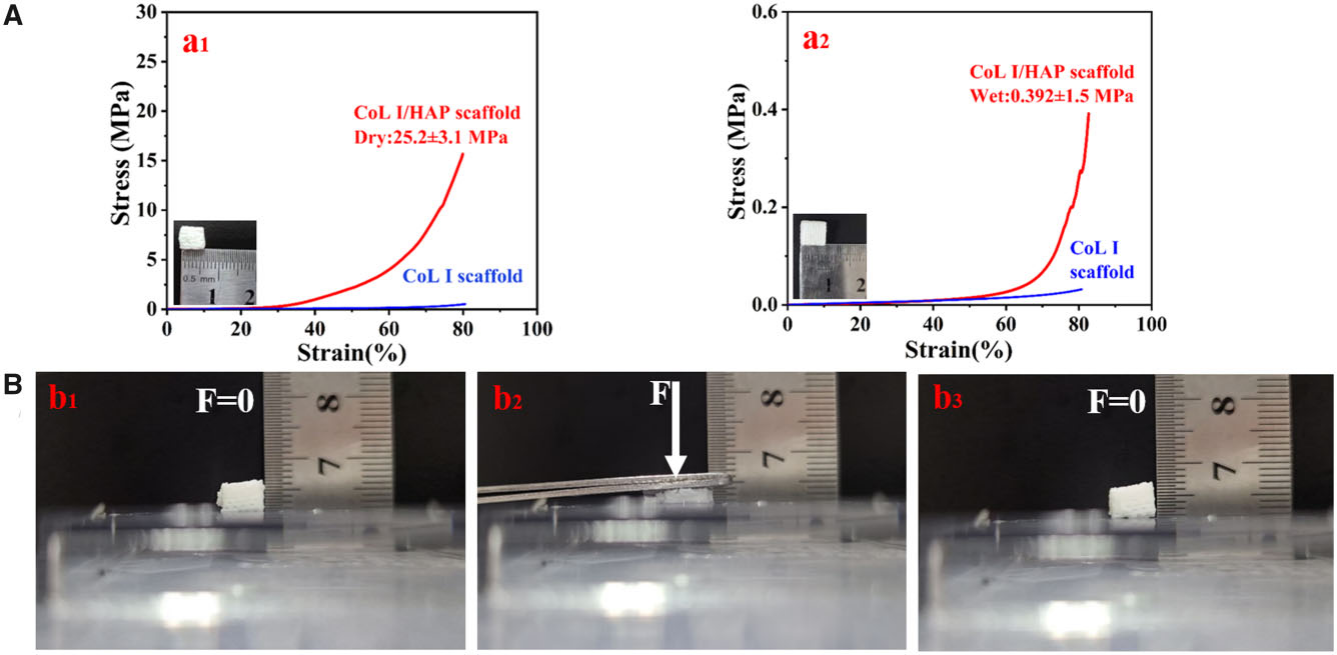
Mechanical properties characterization of scaffolds: (**A**) typical strain–strain curves of dry (a_1_) and wet (a_2_) scaffolds; (**B**) elastic behavior of scaffolds under a strain of 80% ((b_1_) is the initial state of the scaffold; (b_2_) is the stress state of the scaffold and (b_3_) is the state of the scaffold after the force was removed).

Mechanical properties are a crucial parameter for assessing tissue-engineered scaffolds; therefore, we conducted compressive testing on the printed scaffolds. Figure 5A shows the mechanical properties of the lyophilized and wetted scaffolds. The two typical stress–strain curves had the same trend. After compressing to 80% strain, the compressive modulus of the lyophilized and wetted scaffolds was 25.2 ± 3.1 and 0.39 ± 0.11 MPa, respectively. The compression properties of the freeze-dried scaffolds were much greater than that of the wet scaffolds because the scaffolds lost moisture during the freeze-drying process. This has indicated that water content plays an essential role in determining the mechanical properties of collagen fibers [45]. In the absence of water molecules, these sites that bind to water are likely available for intermolecular binding, which can enhance the collagen triple helix and prevent slippage and translation between adjacent molecules [46].

Compared to pure collagen scaffolds, the composite scaffold exhibits significantly improved mechanical properties due to the enhanced phase of HAP [33]. Both moduli of the composite scaffolds greatly exceed those of pure collagen scaffolds. As demonstrated in Figure 5B, these scaffolds also possess excellent elasticity. After applying pressure to the scaffolds, they can recover nearly 100% of their original shape and structure under the maxim strain of 80%. Collagen has good elasticity. Meanwhile, HAP has high strength with less elasticity. After the combination of the two materials in this study, the resulting composite material exhibited a high degree of elasticity, which can be attributed to the typical structure of polymer matrix composites. In this composite, HAP is distributed within the polymer matrix as depicted in Figure 4C, indicating that its deformation shares similar features with collagen matrices.

### *In vitro* viability and osteogenic capacity

**Figure 6.**
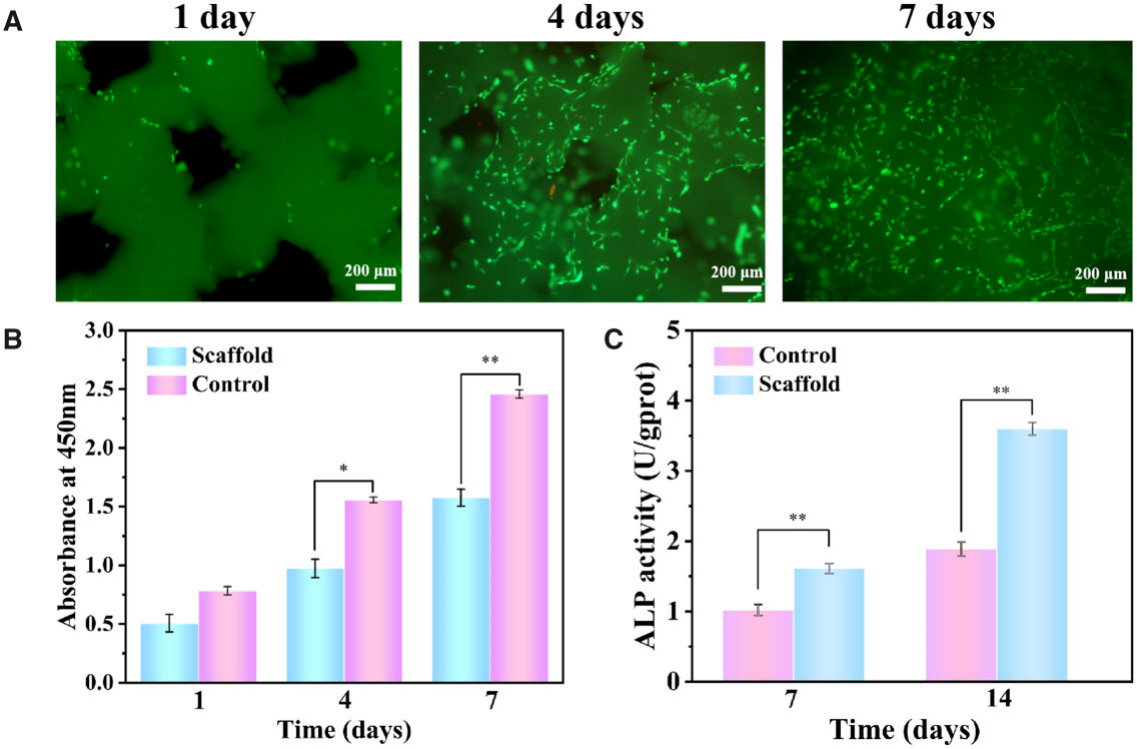
Characterization of biocompatibility and osteogenic properties of scaffolds: (**A**) fluorescence micrographs of cells stained with AM/PI (green, living cells; red, dead cells); (**B**) the OD value of cells cultured on the scaffolds for 1, 4 and 7 days; (**C**) the ALP activity after BMSC cells were cultured on the scaffolds for 7 and 14 days.

To explore whether the composite scaffolds have good cytocompatibility and are suitable for bone tissue repair, we analyzed and evaluated the cytotoxicity of the composite scaffolds by AM/PI live and dead assays, as shown in Figure 6A. On the first day, the total amount of cells on the scaffolds was relatively few. Moreover, only living cells stained green were observed on the scaffolds, and no apoptotic cells stained red were observed, indicating that the scaffolds had extremely low cytotoxicity. After 4 and 7 days of culture, it was observed that a large number of living cells appeared on the scaffolds. The results show that the cells proliferated in the scaffolds, with only a few apoptotic cells. Figure 6B shows the viability of cells on the scaffolds based on OD values. From the data collected on Days 1, 4 and 7, it was observed that the OD values on Day 7 were three times higher than those recorded on Day 1. The results obtained indicate a significant proliferation of cells on the scaffolds.

To assess the osteogenic potential of the composite scaffolds, we conducted an ALP activity assay on them as depicted in Figure 6C. BMSCs were cultured on the composite scaffolds for 7 and 14 days. The results showed a significant increase in ALP secretion compared to the control group. With the increase of culture days, the ALP secretion also increased significantly. This performance shows that the composite scaffolds may possess bioactivity to promote bone formation [26].

### Cell behavior

**Figure 7.**
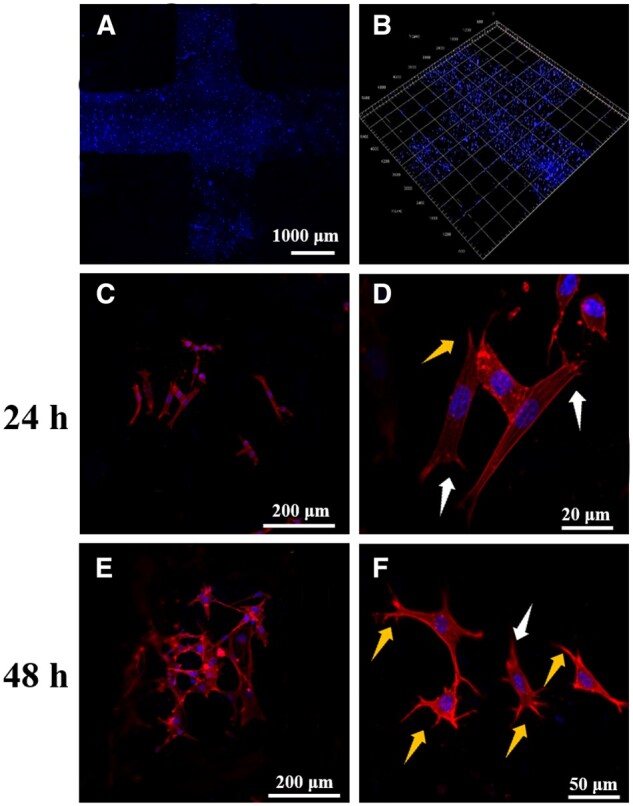
(**A**, **B**) LSCM image of the cell-laden scaffolds. (**C**–**F**) LSCM image of cells spreading behavior at 24 and 48 h (white arrow: the scalloped lamellipodia; yellow arrow: the filopodia).

The printed cell-loaded scaffold was evaluated under LSCM. The nuclei stained blue by DAPI were evenly distributed in the printed scaffolds, as shown in Figure 7. By observing the distribution of the nuclei, the 2D (Figure 7A) and 3D (Figure 7B) images of the scaffolds confirmed that the cells were successfully and homogeneously loaded in the scaffold.

To evaluate composite scaffolds’ cell affinity and adhesion, cells’ spreading and adhesion state on the scaffolds were observed and photographed under an LSCM, as shown in Figure 7C–F. After 24 h of cell growth on the scaffolds, most cells exhibited plate-like pseudopodia. However, after 48 h of culture, a greater number of cells displayed filamentous pseudopodia on the scaffolds [47]. The observation of cell morphology on the composite scaffolds indicated excellent biocompatibility.

The next step will be to further explore the cell viability of the cell-supported scaffold under a more extended period by adjusting the material’s concentration and proportion.

## Conclusions

In this study, the 3D scaffold of HAP/collagen was prepared with a gelatin support bath and the cell-laden 3D printing scaffold. The prepared composite system has rheological properties of shear thinning and viscosity recovery, suitable for extrusion 3D printing. A gelatin support bath supported 3D-printing composite patterns effectively, ensured high fidelity and further realized printing the cell-laden scaffold at room temperature. Combining nano-HAP with collagen endowed the bionic bone 3D scaffold with good mechanical properties. *In vitro* cell evaluation showed that the HAP/collagen 3D scaffold had good cytocompatibility and higher ALP expression. The 3D printing with a gelatin bath provides a way for bio-manufacturing for bone tissue repair. Although the study used a support bath scheme to achieve a cell-laden printing scaffold with bone composition at room temperature, the construction of micro-bone tissue structure is still insufficient by current processing techniques. The fine structure of the scaffold would serve as a microenvironment for guiding cell proliferation, differentiation and tissue regeneration, and still need to be developed via 3D printing technology.

## Supplementary Material

rbad067_Supplementary_Data
