# Supplementary material for: Construction of 3D bioprinting of HAP/collagen scaffold in gelation bath for bone tissue engineering
Source: Regen Biomater. 2023 Aug 11;10:rbad067. doi: 10.1093/rb/rbad067 (PMC10466082; doi:10.1093/rb/rbad067)
Supplement: rbad067_Supplementary_Data [file rbad067_supplementary_data.docx]

**Printing parameters**

The top view of the scaffold is a square whose side length L_1_ = 5.7 mm, the pore width L_2_ = 0.76 mm, the stent height H_1_ = 1.48 mm, the height H_2_ = 0.56 mm, and the fiber diameter is 22 G stainless steel needle diameter R = 0.41 mm. To ensure optimal bonding between each scaffold layer, an interference fit has been incorporated into the contact area with a 0.055 mm overlap. After exporting the model in STL format, it is necessary to slice the model and plan the printing path. The specific parameters for slicing are a layer height of 0.41 mm, an extrusion multiplier of 180, and a processing speed of 1 mm/s.


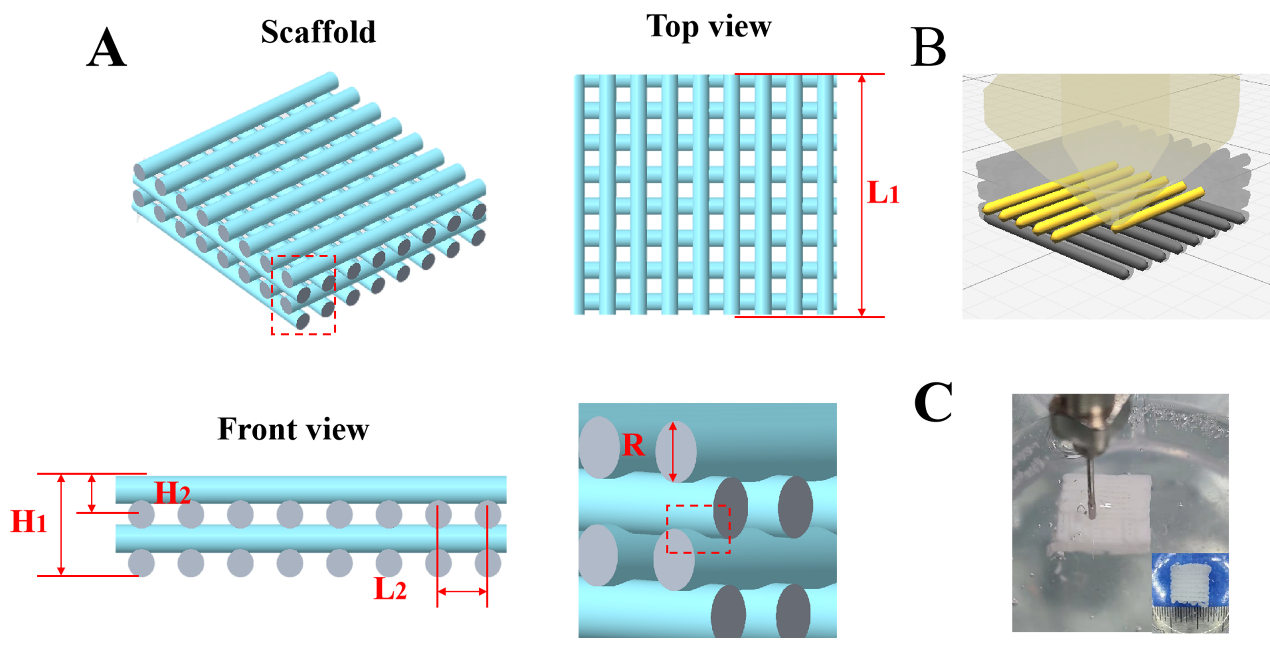


Figure S1. Print schematic: (A) Three-dimensional model of wooden crib porous structure scaffold; (B) schematic diagram of scaffold processing; (C) print the scaffold in the gelatin-supporting bath.

**Preparation of gelatine-supporting bath**

To prepare the gelatin-supporting bath, we utilized sterile water to create an 11 mM calcium chloride (CaCl_2_) solution and stored it at a temperature of 4ºC in a refrigerator. As per literature [1], the pH of the weakly acidic calcium chloride solution ranges from 5-6. Under the condition that -NH_2_ of gelation can be converted to -NH_3_^+^, then -NH_3_^+^ and COO^-^ produce a polyelectrolyte effect together with Ca_2_^+^. All these interactions enhance the gelation bath to support the molding processing (Ref. 1 in supporting information). Subsequently, 15 mL of CaCl_2_ solution was introduced into a 50 mL centrifuge tube, followed by the addition of sterile A-type gelatin particles to generate a 4.5% (w/v) gelatin solution. The centrifuge tube was hermetically sealed with sealing film and incubated in a constant temperature water bath at 60ºC for thirty minutes until complete dissolution of type A gelatin particles occurred. The centrifuge tube was subsequently transferred to a refrigerator at 4ºC for overnight storage. Afterward, 35 mL of CaCl_2_ at 4ºC and 15 mL of gelatin were combined in a beaker and homogenized using a high-speed dispersing homogenizer (IKA, Germany) for 120 seconds. Then, the blended gelatin was loaded into a centrifuge tube and centrifuged at 4200 rpm for 3 min, and the supernatant was removed and replaced with 11 mM CaCl_2_ at 4ºC. The slurry was subjected to vortexing and centrifugation repeatedly until no air bubbles were visible on the surface of the supernatant, indicating the effective removal of most soluble gelatin [2].


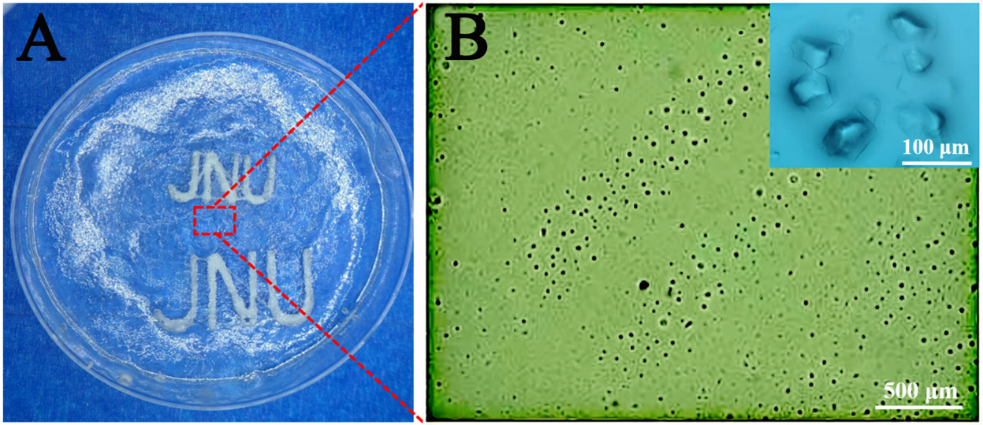


**Figure S2.** Micromorphology of the gelatin-supporting bath: (A) Macro image of gelation bath; (B) microscopic image of gelation bath.

**References**

1. P. Li, T. Wang, J. He, J. Jiang, F. Lei. Synthesis, Characterization and selective dye adsorption by pH-and ion-sensitive polyelectrolyte galactomannan-based hydrogels. *Carbohydrate Polymers* 2021; 264: 118009.

2. T.J. Hinton, Q. Jallerat, R.N. Palchesko, J.H. Park, M.S. Grodzicki, H.-J. Shue, M.H. Ramadan, A.R. Hudson, A.W. Feinberg. Three-dimensional printing of complex biological structures by freeform reversible embedding of suspended hydrogels. *Sci. Adv* 2015; 1: e1500758.
